# Glioblastoma vasculogenic mimicry: signaling pathways progression and potential anti-angiogenesis targets

**DOI:** 10.1186/s40364-015-0034-3

**Published:** 2015-04-18

**Authors:** Jin-ming Mao, Jing Liu, Geng Guo, Xing-gang Mao, Chang-xin Li

**Affiliations:** Department of Neurology, The First Clinical Medical College of Shanxi Medical University, 85# South Jie Fang Road, Taiyuan, Shanxi Province 030001 People’s Republic of China; Department of Neurosurgery, The First Hospital, Shanxi Medical University, 85# South Jie Fang Road, Taiyuan, Shanxi Province 030001 People’s Republic of China; Department of Neurosurgery, Xijing Hospital, Fourth Military Medical University, 15# West Chang Le Road, Xi’an, Shaanxi Province 710032 People’s Republic of China

**Keywords:** Vasculogenic mimicry (VM), Glioblastoma (GBM), Angiogenesis, Cell signaling

## Abstract

Glioblastoma (GBM) is a highly angiogenic malignancy that is resistant to standard therapy; neo-formed vessels of this aggressive malignancy are thought to arise by sprouting of pre-existing brain capillaries. However, the conventional anti-angiogenic therapy, which seemed promising initially, shows transitory and incomplete efficacy. The discovery of vasculogenic mimicry (VM) has offered a new horizon for understanding tumor vascularization. VM is a tumor cell-constituted, matrix-embedded fluid-conducting meshwork that is independent of endothelial cells and is positively correlated with poor prognosis. Therefore, a better understanding of GBM vasculature is needed to optimize anti-angiogenic therapy. This review focuses on the signaling molecules and cascades involved in VM in relation to ongoing glioma research, as well as the clinical translational advances in GBM that have been offered by the development of optimized anti-angiogenesis treatment modalities.

## Introduction

Glioblastoma (GBM) is the most common and lethal malignant brain tumor in adults. It is an extraordinarily aggressive malignancy characterized by extensive micro-vascular proliferation and is highly resistant to intensive combination therapies. The prognosis for GBM patients is extremely poor despite the use of comprehensive treatment involving gross tumor resection, chemotherapy and/or radiotherapy, with an average life expectancy of 12 to 15 months once diagnosed [1,2].

GBM is one of the most vascularized tumors, and its poor prognosis primarily results from its invasive properties. Indeed, an accepted tenet underlying tumor survival is that a blood supply is required to sustain growth and invasion [3]. The neoplastic angiogenesis research ignited by this important premise was the basis for enthusiastic preclinical trials, which ultimately reaped disappointing clinical results [4]. Thus, investigators accepted that tumor perfusion mechanisms are much more sophisticated than we previously realized [2].

**Figure 1:**
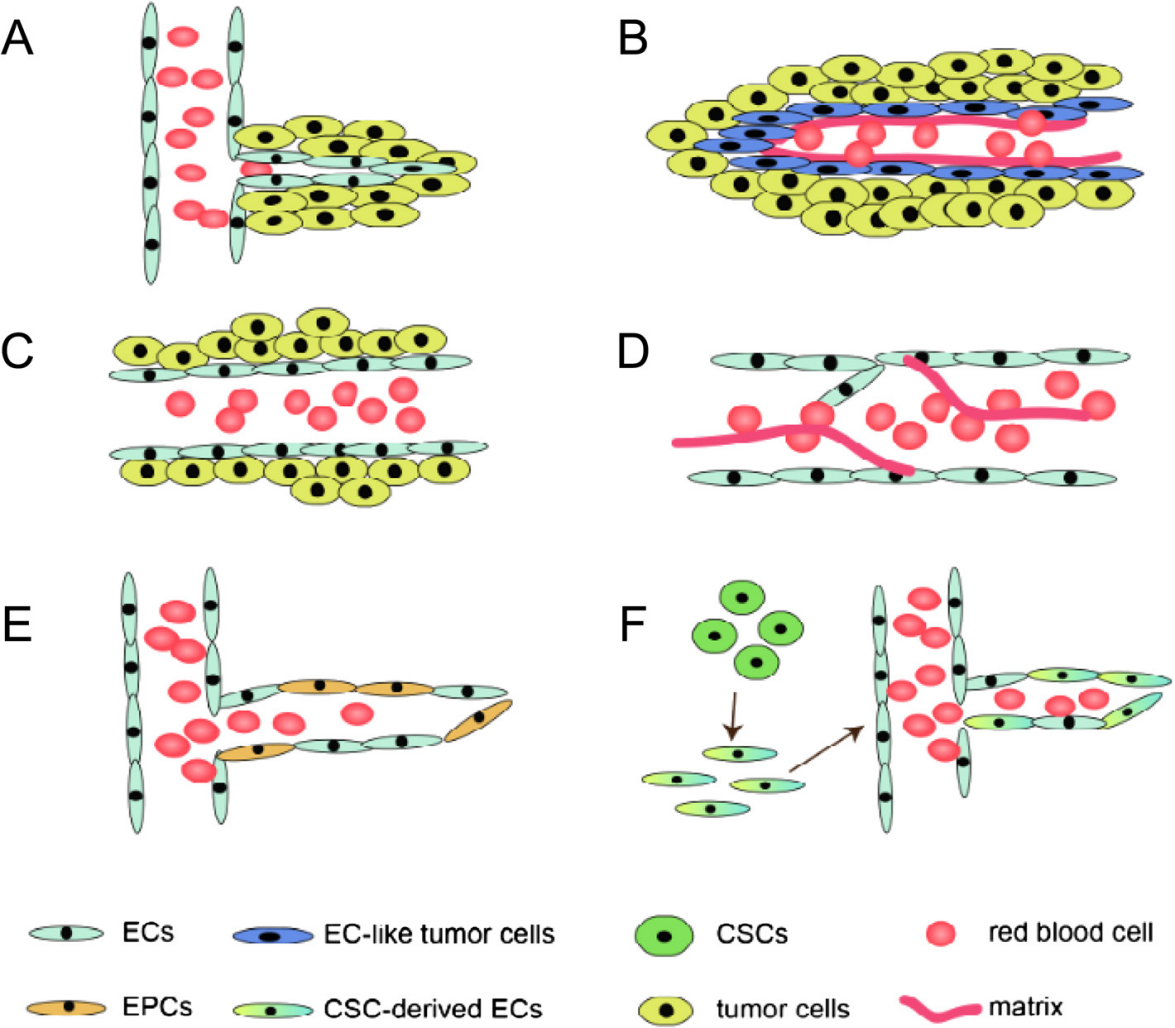
Mechanisms of tumor vascularization. The different mechanisms of tumor vascularization are depicted in the figure. These include: **(A)** sprouting angiogenesis: sprouting of pre-existing brain capillaries through proliferation and migration of local ECs; **(B)** vasculogenic mimicry: tumor cell-constituted, matrix-embedded fluid-conducting meshwork; **(C)** vascular co-option: tumor cells grow along pre-existing blood vessels; **(D)** vascular intussusception: internal division of the preexisting capillary plexus; **(E)** bone marrow-derived vasculogenesis: recruitment of circulating endothelial precursors (EPCs) to form new vessels; **(F)** cancer stem-like cell-derived vasculogenesis: transdifferentiation of CSCs into ECs to form new vessels.

The newly discovered vascular network structure, vasculogenic mimicry (VM), was first described and defined by Maniotis et al. for malignant melanoma in 1999 [5]. The discovery of VM simultaneously sparked intensive controversy [6] and brought a new vision to tumor therapy. VM is defined by a fluid-conducting, matrix-embedded meshwork that is independent of endothelial cells (ECs), but instead is formed by certain types of tumor cells through their acquirement of plasticity to mimic endothelial function [7]. Since the discovery of VM, cumulative studies have contributed new insights into the underlying molecular pathways supporting its existence in a variety of non-melanoma aggressive tumors [8-13], including GBM [14]. CD34 or CD31 and PAS dual-staining have been applied to visualize the EC-free, matrix-rich morphological pattern of VM [5,8-14]; and immunohistochemistry and microarray analysis have been applied to identify its undifferentiated, embryonic-like phenotype [5,7,14]. Two distinctive VM types--tubular type [15] and patterned matrix type [14,16]--have been described. From the extensive literature across this vast field, we now appreciate that the simplistic model of sprouting angiogenesis is far too limited to describe the complex tumor vasculature. There are several other paradigms reported in addition to VM (Figure 1), including vascular co-option, vascular intussusception, bone marrow-derived vasculogenesis and cancer stem-like cell-derived vasculogenesis [3]. For glioma, the phenomenon of VM is associated with high grades of tumor invasiveness and poor prognosis [1,17]. As an alternative to traditional anti-angiogenic therapy, the identification of molecules and signaling pathways relating to VM may offer potential therapeutic targets to improve treatment [18]. In this review, we summarize the current progress in understanding the molecular mechanisms revealed by ongoing glioma VM research and discuss potential VM-targeting strategies for the future development of GBM therapies.

## Functional characterization of VM

Tumors depend on an adequate blood supply for growth and metastasis [2,3]; therefore, intratumoral microvessel density is assessed as a biomarker for tumor progression and is considered a valuable prognostic indicator [19]. However, clinical trials have shown that some tumors with low intratumoral microvessel density have poor prognosis, and conventional anti-angiogenic results are not consistently predictive. The inconsistency between theory and practice suggests that alternative vascular mechanisms exist, which was the basis for proposing VM as a new topic of research [2].

The presence of characteristic VM structures in tumor tissues is associated with poor clinical outcome, suggesting that VM contributes functionally to tumor progression. Blood circulation demonstrated by confocal indocyanine green angiography revealed that VM patterns are not part of the EC-lined vascular system. Fluid enters these channels through leakage and circulates within the VM network rather than accumulating in a pool [20].

In addition, microarray analysis illustrated that tumor cells involved in the VM structure convert to a dedifferentiated, embryonic-like phenotype, which appears to be multipotent to act as either ECs or common tumor cells. Moreover, the co-localization of tumor cells and ECs lining the vascular endothelium (referred to as mosaic vessels [16]) indicates a direct or indirect physical connection between endothelial vessels and VM networks.

## Biological phenomena of Glioma stem-like cells (GSCs) related to VM

Tumor cells capable of VM formation exhibit high plasticity indicative of a multipotent phenotype that resembles embryonic stem cells. This phenomenon is confirmed for cancer stem cells (CSCs). A simple recapitulation of the CSC theory is as follows: genetically dysregulated tumor cells are embryonic-like, therefore “plastic”, and therefore capable of expressing vascular-like phenotypes [16]. Though specific criteria for the identification of CSCs have not been established, screening and enrichment of CSCs prospectively in a series of malignancies has identified stem cell biomarkers such as CD133 and Nestin, which are also expressed in GSCs [21,22]. CSCs can be enriched by the use of anti-CD133 antibodies or through the generation of neurospheres in a certain culture condition, mostly serum-free media containing epidermal growth factor and basic fibroblast growth factor [23]; and GSC screening undergoes similar procedures. As for the origins of GSCs, in addition to the multistep mutations of normal stem cell genomes, GSCs might also be derived partly due to dedifferentiation via the process of epithelial-mesenchymal-transition (EMT), a process about regaining dedifferentiated phenotypes and mesenchymal features [24]. Therefore, GSCs may be considered a bridge between EMT and VM formation.

**Figure 2:**
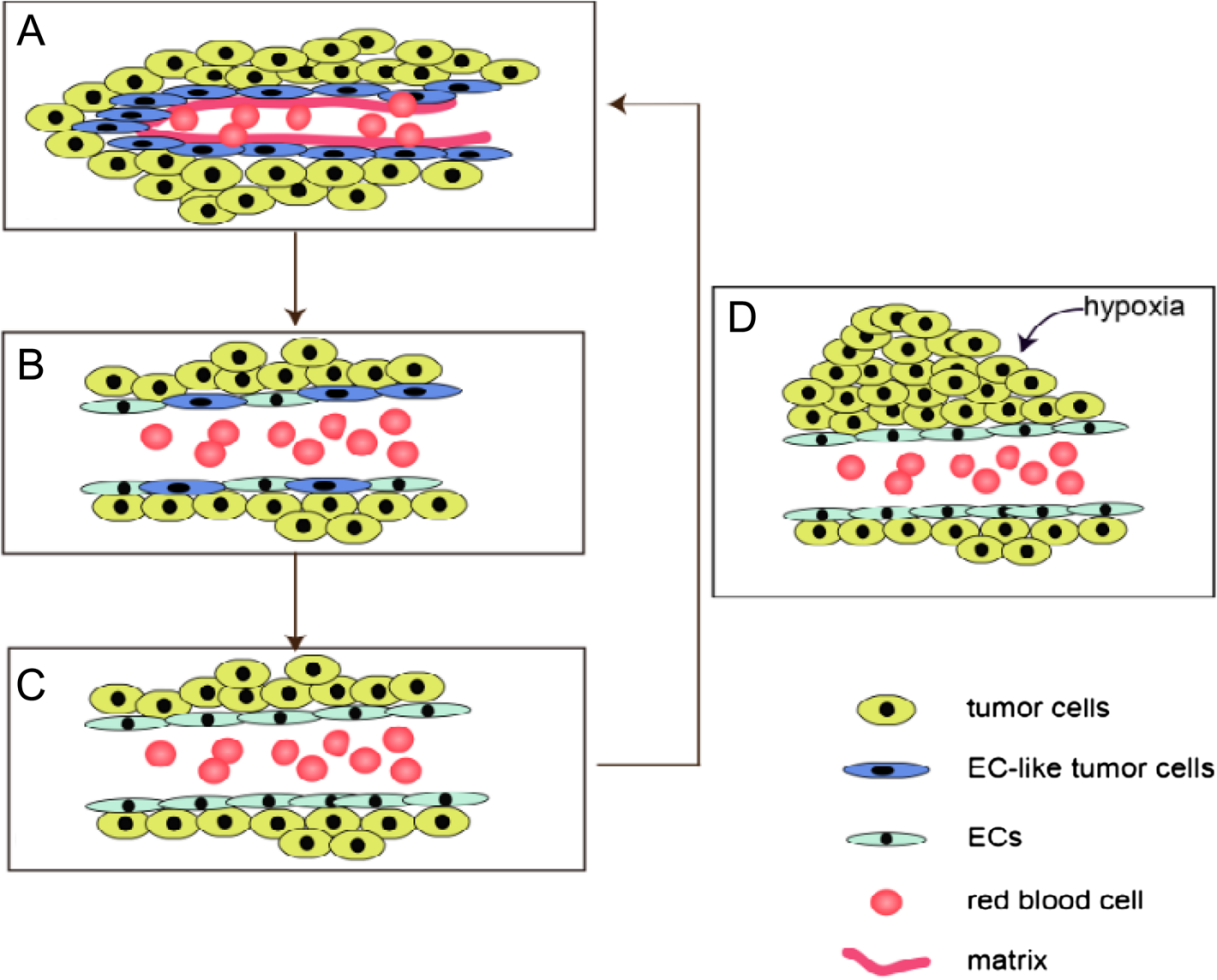
The three stages of tumor blood supply pattern. The possible connection between VM channels and the endothelium-dependent vessels: different stages of a continuous process--**(A)** VM channels, **(B)** mosaic vessels and **(C)** the endothelium-dependent vessels. Figure 2(D)--tumor proliferation acts as a trigger point to anther tumor blood supply cycle.

Similar to normal neural stem cells, GSCs possess the capacity of self-renewal and multi-lineage differentiation; however, aberrant gene regulation confers their tumorigenicity. GSCs participate in VM formation by interacting with the vascular niche to shape the proper tumor microenvironment and by differentiating into EC-like tumor cells to constitute VM structure. The cells derived from GSCs present multi-phenotype features of different lineages, such as neural cells and mural cells, thus effectively mimicking the microcirculatory system. The tumor vasculature of GSCs in an incomplete stage is thought to be formed by VM channel [23], consistent with the three stages of tumor blood supply pattern proposed by Zhang et al. [25], namely VM channels, mosaic vessels and the endothelium-dependent vessels (Figure 2).

## Signaling cascades involved in VM

There are two main aspects that underlie the formation of VM, i.e. highly plastic tumor cells and the tumor microenvironment. The tumor cells lining the vasculature display an undifferentiated embryonic-like biological and molecular phenotype [26], suggesting the involvement of CSCs. CSCs are a subpopulation of tumor cells that possess the capacity of self-renewal, multi-lineage differentiation, tumor initiation and resistance to chemo- or radio-therapy [27]. Additionally, the tumor microenvironment is a critical factor that conventional tumor cell-targeted therapy fails to take into account. Changes in the extracellular microenvironment may persist after removal or destruction of an aggressive tumor, thus resulting in the recurrence or continuance of certain tumors [28]. The over-expression of VEGFR-2 [21,26,29], EphA2 [30], VE-cadherin [31], MMPs and TGF-β [32], among other molecules, have been demonstrated to be closely associated with vasculature formation. Hypoxia, which can be induced by Bevacizumab therapy [33], represents an additionally factor that might partially activate remodeling of the extracellular microenvironment as a result of oxygen-glucose deprivation.

**Figure 3:**
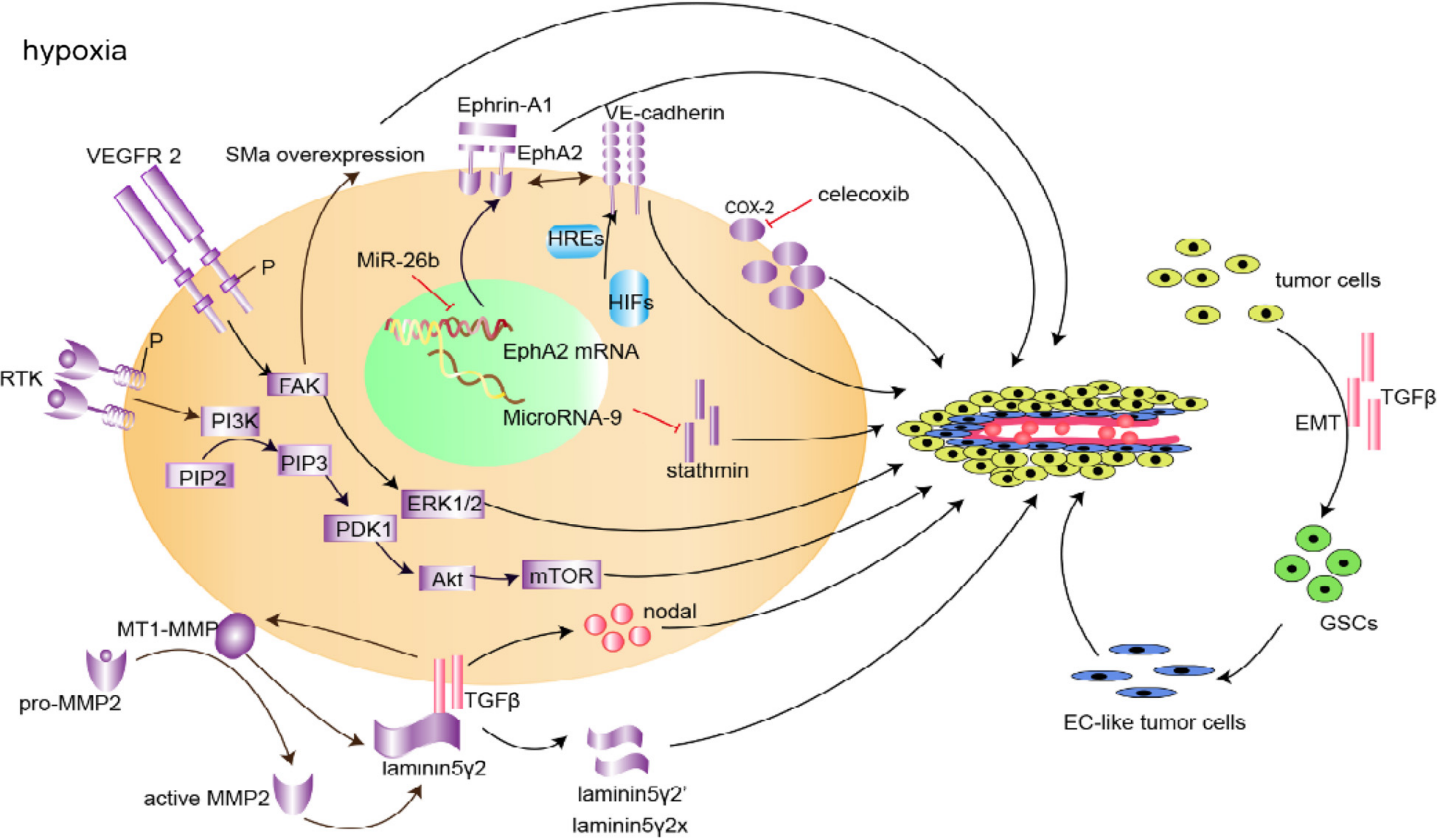
Schematic model of signaling pathways implicated in glioma vasculogenic mimicry (VM). The whole VM process proceeds under the condition of hypoxia. Only signaling molecules which have been specifically modulated using small inhibitory RNAs, blocking antibodies or small molecule inhibitors are depicted -- demonstrating their ability to directly or indirectly affect VM, and are categorized as microenvironment (purple), embryonic/stem cell (red), and hypoxia signaling pathways (blue). There are no specific boundary lines between the three parts and overlap between major VM signaling pathways demonstrated coordinated work of these pathways.

The multipotent phenotype underlying VM is supported by a complex network of potential signaling pathways, and an increasing number of studies have been conducted to illustrate the fundamental mechanisms in order to establish new treatment regimens. We present some of the signaling pathways attributed to VM formation in GBM (Figure 3, Table 1) according to their association with the embryonic/stem cell phenotype (section Stem cell pathway of GBM), the glioma microenvironment (section Glioma microenvironment-related signaling pathways) and hypoxic conditions (section Hypoxia-related mechanism of VM).

**Table 1:** **Brief summary of the potential molecular targets for optimized anti-angiogenesis therapy**

**Project**	**Potential molecular targets**	**Related process**	**Tumor types/cell lines**	**method**	**PMID**
**Signaling pathway**					
TGFβ Signaling pathway	TGFβ	EMT-embryonic phenotype regain;	U251	Transfection and RNAi	22104964
		tumor microenvironment remodulation	SHG44		24370825
VEGFR-2/Flk-1 pathway	VEGFR-2	Self-renewal, tumorigenicity and tubular formation	U87 derived GSCs,	Transfection and RNAi;	23536763
			GBM samples	Immunohistochemistry,	
				Xenograft models	
			U87	RNAi;	22654102
			GSDC	xenotransplantation	
VE-cadherin/CDH5 and EphA2 pathways	VE-cadherin	hypoxia induced microenvironment remodulation;	Glioma samples;GSCs	RT-PCR, western blot, immunohistochemistry;	23645533
				RNAi and xenograft models	
	EphA2 and MiR-26b	Cell proliferation, invasion and tubular formation	Glioma samples;	RT-PCR;	21264258
			U251 and C6	Transfection and RNAi	
RTK/PI3K/Akt/mTOR pathway	RTK/PI3K/mTOR	Cell proliferation, apoptosis, microenvironment remodulation.	U251 and T98G	Pharmacologic inhibitor, RNAi	24418474
MMP-laminin5γ2 chain pathway	MMPs	a shared downstream signaling pathway	U251	Transfection and RNAi	22104964
			SHG44		
others	COX-2	Promote cell survival, proliferation, and angiogenesis and prohibit apoptosis	Glioma specimen	Immunohistochemistry, Kaplan–Meier survival analysis and log-rank tests	21533525
	MiR-9	Cell proliferation and apoptosis	U87MG	Transfection and RNAi	24043603
			U251		
			SHG44		

### Stem cell pathway of GBM

Tumor cells capable of VM formation exhibit a high plasticity indicative of a multipotent phenotype resembling embryonic stem cells. The in vitro tubular formation capacity and unique gene expression signature of highly invasive melanoma cells suggests that tumor cell VM activity is associated with distinctive genetic dysregulation [5]. Microarray analysis of VM-positive tissues revealed increased expression of genes associated with an undifferentiated embryonic-like phenotype [7]. GSCs participate in VM formation in several ways: they interact with the vascular niche to shape the proper tumor microenvironment [26,32,34] and differentiate into EC-like tumor cells to constitute VM structure [21,22]. Several molecules associated with anaplastic properties of tumor cells are over-expressed in CSCs and are associated with VM formation, including transforming growth factorβ (TGFβ) [32], Nodal [34], and vascular endothelial growth factor receptor-2 (VEGFR-2) [26]. TGFβ and Nodal are members of the TGFβ superfamily and are discussed in section The TGFβ signaling pathway below, while VEGFR-2 is discussed in section The vascular endothelial growth factor receptor-2 (VEGFR-2)/Flk-1 pathway on the basis of its more well-established role in affecting the tumor microenvironment.

#### The TGFβ signaling pathway

The TGFβ superfamily of growth factors is a group of ubiquitous multifunctional cytokines that regulate a plethora of cellular activities, including proliferation, differentiation, migration and survival. Both TGFβ and Nodal are critical members of this family [35]. TGFβ is named after its function in inducing EMT [34], a process whereby fully differentiated epithelial cells divert to a dedifferentiated state and acquire mesenchymal features. TGFβ regulates two different aspects of VM formation: the maintenance of the undifferentiated embryonic state of stem cell via EMT [36] and the induction of tubular formation through modulation of the tumor microenvironment. Studies of breast cancer and melanoma show that TGFβ stimulates tubular formation by regulating MMP expression in epithelial tumors [37,38]. Thus, TGFβ enhances or reduces the activity of the MMP-laminin 5γ2 chain signaling pathway (discussed further in section The MMP-laminin 5γ2 chain signaling pathway). Similar regulatory mechanisms have been detected in GBM [32].

Ling, et al. demonstrated that TGFβ levels correspond with glioma malignancy in vitro. Plasmid transfection of TGFβ mRNA activates the dose-dependent expression of VM-related molecules in glioma cell lines as assessed by RT-PCR, and the activity of MMP2 and MMP9 as assessed by gelatin zymography. Furthermore, inhibition of TGFβ results in a decline in both the quantity and activity of MT1-MMP, which in turn reduces MMP2 activation, thereby impairing tubular formation. EphA2 was not modulated in response to TGFβ regulation in this study, and VE-cadherin (CDH5), which can be induced by potent down-regulation of TGFβ through EMT, was detectably absent [32]. This is inconsistent with the role for VE-cadherin in VM reported by Mao, et al. [31]. A possible explanation for this discrepancy involves the use of glioma cell lines versus GSCs. The putative roles for VE-cadherin and EphA2 in regulating the glioma microenvironment are discussed in further detail below (section The VE-cadherin (CDH5) and EphA2 pathways).

Nodal, a potent embryonic morphogen in the TGFβ superfamily, is a biomarker that normally loses expression during the differentiate process, but becomes aberrantly re-expressed in highly aggressive tumors. Nodal contributes to tumor progression and plasticity through a variety of mechanisms [39]. Though the role for Nodal has not precisely been demonstrated for GBM, the activation of Nodal expression by TGFβ has been shown to promote glioma cell growth [34]. Given the above information, we can presume that both the activation of EMT and the TGFβ-induced expression of MMPs and Nodal play a role in VM formation.

### Glioma microenvironment-related signaling pathways

Pathways known to modulate the glioma microenvironment include the vascular endothelial growth factor receptor-2 (VEGFR-2)/Flk-1 pathway (section The vascular endothelial growth factor receptor-2 (VEGFR-2)/Flk-1 pathway), VE-cadherin (CDH5) and EphA2 pathways (section The VE-cadherin (CDH5) and EphA2 pathways), the RTK/PI3K/Akt/mTOR signaling pathway (section The RTK/PI3K/Akt/mTOR signaling pathway), and the MMP-laminin 5γ2 chain signaling pathway (section The MMP-laminin 5γ2 chain signaling pathway). The potential role of other related molecules in determining the glioma microenvironment in VM is discussed in section Other molecules that regulate the glioma microenvironment in VM.

#### The vascular endothelial growth factor receptor-2 (VEGFR-2)/Flk-1 pathway

VEGFR-2 has emerged as an essential angiogenic mediator of signaling cascades induced by vascular endothelial growth factor (VEGF). High levels of both VEGFR-2 and VEGF are co-expressed during angiogenesis. The VEGF-VEGFR pathway and its downstream molecular cascades are thought to activate both traditional angiogenesis and VM in some aggressive tumors [18,40]. VEGFR-2, a receptor tyrosine kinase, is the earliest differentiation marker for ECs. Its expression in adults is normally restricted primarily to ECs, but is up-regulated transiently during angiogenesis [29]. VEGFR-2 has been reported to be over-expressed in GBM by GSCs at both the mRNA and protein level. Once activated by VEGF, this signaling pathway mediates chemotaxis, self-renewal, tumorigenicity, tubule formation and over-expression of critical VM markers [26]. VEGFR-2 phosphorylation at specific tyrosine residues in an intracellular kinase domain in its C-terminus subsequently activates down-stream intracellular signaling molecular cascades, including the focal adhesion kinase (FAK) and mitogen-activated protein kinase (MAPK)- extracellular signal-regulated protein kinases 1 and 2 (ERK1/2) cascades, and smooth muscle actin (SMa) expression. Activation of the FAK and MAPK-ERK1/2 signaling pathways then mediate cell proliferation, migration, and tubule formation [26]. However, these events do not require VEGF stimulation in GBM-derived tumor cell lines [26,29], which might partially be explained by the constitutive phosphorylation of VEGFR-2 in GSCs [26]. VEGFR-2 also may be activated indirectly by other factors in the tumor microenvironment, either through binding to membrane-associated integrins or by coordinate induction of integrins, leading to increased VEGFR-2 activation [26]. Follow-up studies using animal tumor models also validate the indispensable role of VEGFR-2, which is independent of VEGF and regulates mural-like tumor cell-associated VM in GBM [21,26].

#### The VE-cadherin (CDH5) and EphA2 pathways

VE-cadherin, a member of the cadherin-family, is a transmembrane glycoprotein that is thought to be specifically expressed in ECs. VE-cadherin promotes homotypic cell-cell interactions and was one of the first molecules identified as an important player in VM for melanoma [41]. The Eph family is the largest family of receptor tyrosine kinases (RTKs) and regulates cell proliferation, migration and angiogenesis. Similar to the findings for melanoma [42], both VE-cadherin and EphA2 are expressed at higher levels in VM-positive glioma than VM-negative glioma, and the expression of these genes correlates with the glioma grade and is known to be required for VM network formation [30,31,43]. Low expression levels in glioma cell lines that are incapable of VM formation further indicates a role for these molecules in VM [31,32]. Additionally, knockdown of these genes with short hairpin RNA causes a predominant decrease in VM formation [31,42]. For hypoxia-induced VM, VE-cadherin is up-regulated by hypoxia-inducible factor (HIF) 2α and 1α through direct binding to the VE-cadherin promoter [31]. Furthermore, EphA2 over-expression couples with reduced miR-26b expression, properly speaking, miR-26b down-regulates the levels of endogenous EphA2 protein by binding to a specific microRNA response element in its 3’UTR [30]. In 2006, Hess et al. demonstrated that in VM-positive melanoma, VE-cadherin co-localizes with EphA2 at areas of cell-cell contact, and these two molecules interact either directly or indirectly during the process of VM formation [42]. VE-cadherin regulates EphA2 activity by mediating its auto-phosphorylation through interaction with its membrane bound ligand, Ephrin-A1. Phosphorylated EphA2 subsequently regulates the phosphoinositide 3-kinase (PI3K) and FAK [44,45] pathways, thus activating the PI3K-MMP-laminin 5γ2 chain signaling pathways and related intracellular signaling cascades. Whether a similar pathway of regulation occurs in GBM awaits further verification.

#### The RTK/PI3K/Akt/mTOR signaling pathway

The mutant RTK/PI3K/Akt/mTOR pathway is the most frequently deregulated signaling cascade in GBM and regulates various cellular processes such as proliferation, growth, apoptosis, and cytoskeletal rearrangement [46]. This pathway has been accepted as a novel genetic target for acquired glioma resistance [45,47,48].

RTKs are the largest group of the enzyme linked receptor families, which possess an N-terminal extracellular ligand-binding domain, a single anchoring transmembrane-helix, and a cytosolic C-terminal region that contains the catalytic domain [46]. In glioma, EGFR is one of the most prominent members [46,47]. PI3K is a cytoplasmic lipid kinase consisting of a regulatory subunit, p85, and a catalytic subunit, p110. Combination of p85 and the RTK results in the activation of catalytic subunit (p110), which then catalyzes the phosphorylation of PI 3,4-bisphosphate (PIP2) into 3,4,5-triphosphate (PIP3) [46]. PIP3 in turn activates phosphoinositide-dependent kinase-1 (PDK1), which subsequently activates Akt by phosphorylating specific amino acid sites. Activated Akt regulates a series of downstream molecules such as mTOR, which controls a variety of cellular functions [46]. Deficiency of PTEN and related regulators in glioma, which serve as negative regulators of PIP2 and PIP3, promotes tumor invasiveness [49]. Conversely, targeting of the mutant RTK/PI3K/Akt/mTOR signaling pathway leads to consistent reduction in the invasion and migration by VM channels in U251 glioma cells [49]. In concert with these results, VM-related molecules such as EphA2 and MMPs also show a corresponding reduction [49]. These findings suggest that the aberrant RTK/PI3K/Akt/mTOR pathway plays a role in GBM VM formation.

#### The MMP-laminin 5γ2 chain signaling pathway

The laminin 5γ2 chain is the main component of the basal membrane, which can be degraded by MMPs. The over-expression of MMPs in several malignant tumors such as aggressive melanoma [50] and GBM [17,32] can promote tumor invasion and migration. In GBM cell lines [32] and GBM samples [17], MMPs showed a positive correlation with VM formation.

The MMP-laminin5γ2 chain pathway has been suggested as a common downstream signaling pathway of several molecular cascades. The MMP-laminin 5γ2 chain and other signaling regulators associated with VM (TGFβ [32], VE-cadherin, EphA2, PI3K [42] etc.) are coordinately over-expressed in a number of malignancies including GBM [32] and aggressive melanoma [50]. MMP transcription is regulated by upstream regulators such as TGFβ [32]. When activated by upstream molecules, MT1-MMP converts proMMP2 to active MMP2. Both MT1-MMP and MMP2 promote the cleavage of the laminin 5γ2 chain into promigratory γ2′and γ2x fragments, which in turn stimulate migration, invasion, and VM formation [32]. MT1-MMP siRNA promotes a sharp decrease in the cleavage of the laminin 5γ2 chain and VM formation [32]. Thus, the MMP-laminin5γ2 chain pathway may serve as the final executor of VM formation for several molecular cascades.

#### Other molecules that regulate the glioma microenvironment in VM

Cyclo-oxygenase-2 (COX-2), an inducible isoform of the prostaglandin synthesis enzyme cyclooxygenase, shows a positive association with VM channel formation [51]. Pathophysiological factors induce the expression of this enzyme, which is not constitutively expressed in physiological states. COX-2 has been shown to promote cell survival, proliferation, and angiogenesis and prohibit apoptosis, all of which are involved in tumor progression [51]. Recent reports have revealed that highly invasive human glioma cell lines exhibit higher COX-2 expression with vascular channels formation when cultured on three-dimensional Matrigel, whereas non-invasive cell lines do not exhibit this biological phenomenon. These results are supported by studies of human glioma specimens [17]. Inhibition of COX-2 with Celecoxib or specific siRNAs caused a noteworthy reduction in VM formation, suggesting that COX-2 functions in the formation of VM structures.

Other molecules potentially associated with VM formation in GBM include microRNA-9 (miR-9) and Galectin-1 (Gal-1). MiR-9, a tissue-specific microRNA in the central nervous system, inhibits VM formation of glioma cell lines by suppressing Stathmin expression [52]. Gal-1 is regulated by a brain-expressed X-linked gene and is reported to be associated with VM in vitro and in vivo in an oligodendroglioma model [53]; therefore, a similar role for Gal-1 might characterize GBM. Though the exact mechanisms of these molecules are not well elucidated, it is likely that these so-called VM-related pathways co-operate with other pathways in the remodeling of the VM extracellular microenvironment.

### Hypoxia-related mechanism of VM

Hypoxia may potentially be the earliest inducer of VM and influences VM throughout the process. Hypoxia is more easily detectable in more invasive and rapidly proliferating tumors. Furthermore, the role for hypoxia in VM is supported by findings that in vitro hypoxic conditions can lead to VM formation [31]. Induction of VM formation in the absence of oxygen may explain the poor outcome of conventional anti-angiogenic therapy in aggressive melanoma [2]. As a hallmark of most tumors, hypoxia participates in different pathways to maintain a stem cell-like phenotype, regulate cellular differentiation, and promote tumor invasion, metastasis, resistance to apoptosis, angiogenesis and VM [18].

During VM formation in GBM, HIF2α and 1α regulate VE-cadherin expression by directly interacting with hypoxia responsive elements (HREs) in its promoter [31]; however, in aggressive melanoma, though hypoxia induces VM via VE-cadherin, VE-cadherin is up-regulated by Bcl-2 rather than HIFs [54]. Moreover, during the process of GSC generation and transdifferentiation towards tubular formation in vitro, hypoxia offers an indispensable condition for GSCs to differentiate into a group of specialized cells that express specific biomarkers [55], many of which are thought to be promoted by HIFs [54]. Though much work has been done on VM formation, the substantial cellular and molecular mechanisms underlying the effects of hypoxia in the pathogenesis of VM in GBM remain largely elusive.

## Clinical-translational advances in the treatment of GBM and future prospects of VM as a therapeutic target

As highlighted by the multitude of signaling pathways discussed above, comprehensive treatments are indispensable for the control of highly aggressive tumors. Consequently, the understanding of how mono-therapies coordinate to optimize therapeutic outcomes may help to further the understanding of GBM. To achieve this goal, mounting efforts have focused on identifying the related mechanisms. The highly plastic phenotype, sufficient blood perfusion and an adequate nutrient supply are critical for sustaining continual progression of neoplasms. However, the regulatory mechanisms that create these conditions may provide the key to control the process: the tumor cells, along with their stem cells and pericytes, construct their habitat with the assistance of autocrine or paracrine signals, which in turn influence the tumor properties. To obtain the maximum neoplastic effect, a tumor must implement all of the above strategies for the positive reinforcement of growth. Hence, the available tumor therapies, on one hand, are aimed at targeting tumor cells and their progenitor CSCs; and on the other hand, are aimed at simultaneously suppressing the remodeling of the microenvironment and related molecules. For optimal design of therapeutics, the removal or disruption of tumor-related cells, the elimination of essential oxygen and energy supply and the intervention of underlying signaling cascades should all be taken into consideration.

Aside from tumor cell-targeting therapy, the mechanisms of angiogenesis and vascularization are perplexing enough to elicit detailed interpretation. Because the conventional anti-angiogenesis strategies exhibited disappointing results in cumulative clinical trials, several other paradigms about tumor blood supply have emerged, including VM, which has drawn intense controversy since it was first described in 1999. Since then, evidence has begun to accumulate to validate the existence and significance of this novel circulatory structure [1,17,56,57], and meta-analysis has validated the association between VM and poor prognosis [58]. A variety of staining methods from physicochemical dyeing to immunofluorescence techniques have been utilized to reveal the histological features and origins of VM; multidisciplinary approaches have been applied for understanding the aberrant gene transcription and protein translation associated with VM. On the basis of these studies, the functions of a number of genes have been validated in VM, and several molecular pathways have been demonstrated that could provide potential targets for therapy.

Among the potential therapies for VM, anti-angiogenesis by the VEGF mono-antibody Bevacizumab showed minimal efficacy and enhanced tumor invasiveness triggered by hypoxia induction, which may be partially due to VM [33]; and the potent angiogenesis inhibitor Endostar did not affect GBM VM formation [59]. While therapies aimed at classical anti-angiogenesis have shown limited effects, the VM associated mechanisms offered new insights. A handful of preclinical studies suggest that specific compounds affecting components of the previously described vascular, embryonic or hypoxia pathways in tumor cells can inhibit VM formation in xenograft tumor models. VEGFR-2 kinase inhibitors SU1498 and AZD2171 have been shown to reduce VM channel formation in GBM cell lines in vitro and in vivo, concurrent with a reduction in chemotaxis, proliferation and tumorigenicity [26]. Likewise, the MMP inhibitor chemically modified tetracycline [28] and the TGF inhibitor isoxanthohumol [60] downregulate VE-cadherin, EphA2, laminin5γ2 and MMPs and impair VM formation. Lidamycin suppresses tubular structure in a dose-dependent manner, potentially through an apoptosis-related mechanism [61]; while targeting of the RTK/PI3K/Akt pathway enhances the cytotoxic effect of radiation and temozolomide in malignant glioma cells [49].

The increasing abundance of targeted techniques, such as gene knockdown technology, delineates a tangible realization of the potential for counteracting tumor-related mechanisms, which should eventually lead to effective treatments for GBM and other human diseases. In addition, newly devised targeted drug delivery systems circumvent multidrug resistance and demonstrate an enhanced chemotherapeutic efficacy [62,63] . The use of strategies targeting multiple signaling pathways in a combinatorial manner may lead to increased therapeutic efficiency; and studies on VM as a novel and distinct circulatory system will certainly contribute significantly to the future development of anti-tumor treatment regimens.

## Abbreviations

GBM: Glioblastoma
VM: Vasculogenic mimicry
ECs: Endothelial cells
PAS: Periodic acid-schiff
GSCs: Glioma stem-like cells
CSCs: Cancer stem cells
EMT: Epithelial-mesenchymal-transition
TGFβ: Transforming growth factor β
VEGFR-2/Flk-1: Vascular endothelial growth factor receptor-2
MMPs: Matrix metalloproteinases
MT1-MMP: Membrane-type 1
PDK1: Phosphoinositide-dependent kinase-1
FAK: Focal adhesion kinase
MAPK: Mitogen-activated protein kinase
ERK1/2: Extracellular signal-regulated protein kinases 1 and 2
RTKs: Receptor tyrosine kinases
EGFR: Epidermal growth factor receptor
PI3K: Phosphoinositide 3-kinase
HIF: Hypoxia-inducible factor
COX-2: Cyclo-oxygenase-2
HREs: Hypoxia responsive elements
miR-9: microRNA-9
Gal-1: Galectin-1
